# Risk prediction of integrated traditional Chinese and western medicine for diabetes retinopathy based on optimized gradient boosting classifier model

**DOI:** 10.1097/MD.0000000000040896

**Published:** 2024-12-20

**Authors:** Li Xiao, Lixuan Tang, Wenxuan Kuang, Yijing Yang, Ying Deng, Jing Lu, Qinghua Peng, Junfeng Yan

**Affiliations:** a School of Chinese Medicine, Hunan University of Chinese Medicine, Changsha, China; b School of Medicine, Hunan University of Chinese Medicine, Changsha, China; c Xiangtan Chinese Medicine Hospital, Xiangtan, China; d Hunan Provincial Key Laboratory for Prevention and Treatment of Ophthalmology and Otolaryngology Diseases with Chinese Medicine, Hunan University of Chinese Medicine, Changsha, China; e Hunan Provincial Engineering and Technological Research Center for Prevention and Treatment of Ophthalmology and Otolaryngology Diseases with Chinese Medicine and Protecting Visual Function, Hunan University of Chinese Medicine, Changsha, China; f School of Informatics, Hunan University of Chinese Medicine, Changsha, China.

**Keywords:** diabetic retinopathy, gradient boosting machine, integrated traditional Chinese and western medicine, machine learning, risk prediction

## Abstract

In order to take full advantage of traditional Chinese medicine (TCM) and western medicine, combined with machine learning technology, to study the risk factors and better risk prediction model of diabetic retinopathy (DR), and provide basis for the screening and treatment of it. Through a retrospective study of DR cases in the real world, the electronic medical records of patients who met screening criteria were collected. Moreover, Recursive Feature Elimination with Cross-Validation (RFECV) was used for feature selection. Then, the prediction model was built based on Gradient Boosting Machine (GBM) and it was compared with 4 other popular machine learning techniques, including Logistic Regression (LR), K-Nearest Neighbors (KNN), Random Forest, and Support Vector Machine (SVM). The models were evaluated with accuracy, precision, recall, F1 score, and area under the curve (AUC) value as indicators. In addition, grid search was used to optimize the model. To explain the results of the model more intuitively, the Shapley Additive exPlanation (SHAP) method was used. A total of 9034 type 2 diabetes mellitus (T2DM) patients meeting the screening criteria were included in this study, including 1118 patients with DR. 19 features were selected using RFECV in the model construction. We constructed 5 commonly used models, including GBM, LR, KNN, Random Forest, and SVM. By comparing model performance, GBM has the highest accuracy (0.85) and AUC value (0.934), which is the best prediction model. We also carried out hyperparameter optimization of grid search for this model, and the model accuracy reached 0.88, and the AUC value increased to 0.958. Through SHAP analysis, it was found that TCM syndrome types, albumin, low density lipoprotein, triglyceride, total protein, glycosylated hemoglobin were closely related to the increased risk of DR. It can be concluded that TCM syndrome type is the risk factor of DR. The GBM classifier based on grid search optimization, with relevant risk factors of TCM and western medicine as variables, can better predict the risk of DR.

## 1. Introduction

Diabetic retinopathy (DR), which is one of the most prevalent microvascular complications of diabetes mellitus (DM), is now the primary culprit of adult-onset blindness. With the increasing incidence of DM, the prevalence of DR is still rising.^[1]^ With increasing DR trends worldwide, prevention, treatment and associated costs due to DR are a global public health concern. The pathogenesis of this disease is very complex, and western medicine (WM) treatment methods have limited efficacy and long-term side effects. It has been proved that traditional Chinese medicine (TCM) has good clinical effect on the prevention and treatment of DR,^[2–4]^ so international scholars pay more and more attention to it.

The theory of “preventive treatment of disease” is a characteristic of TCM, which emphasizes prevention and early intervention of diseases. It is consistent with the shift in the focus of WM from tertiary DR treatment to secondary prevention strategies for screening and monitoring disease progression.^[4]^ The fact that TCM can effectively prevent DR shows that TCM can accurately make early diagnosis. TCM diagnosis includes the diagnosis of TCM diseases and TCM syndrome types. TCM syndrome type is a generalization of the pathological nature of the body at a certain stage in the course of disease development, including etiology, disease location, disease nature, etc. Theoretically, TCM syndrome type is the indication of DR.

However, the current screening for DR mainly relies on regular fundus examinations, including fundus photography, optical coherence tomography, fluorescein fundus angiography, etc. But these examinations cannot accurately capture small peripheral lesions, and there is an issue of excessive economic burden. Combined with mathematical model, it is an urgent need to realize the risk prediction of DR. At present, some scholars^[5–8]^ have carried out relevant research, but mainly aimed at some biochemical indicators of WM. Improvements are also needed in the approach to model building.

**Figure 1.**
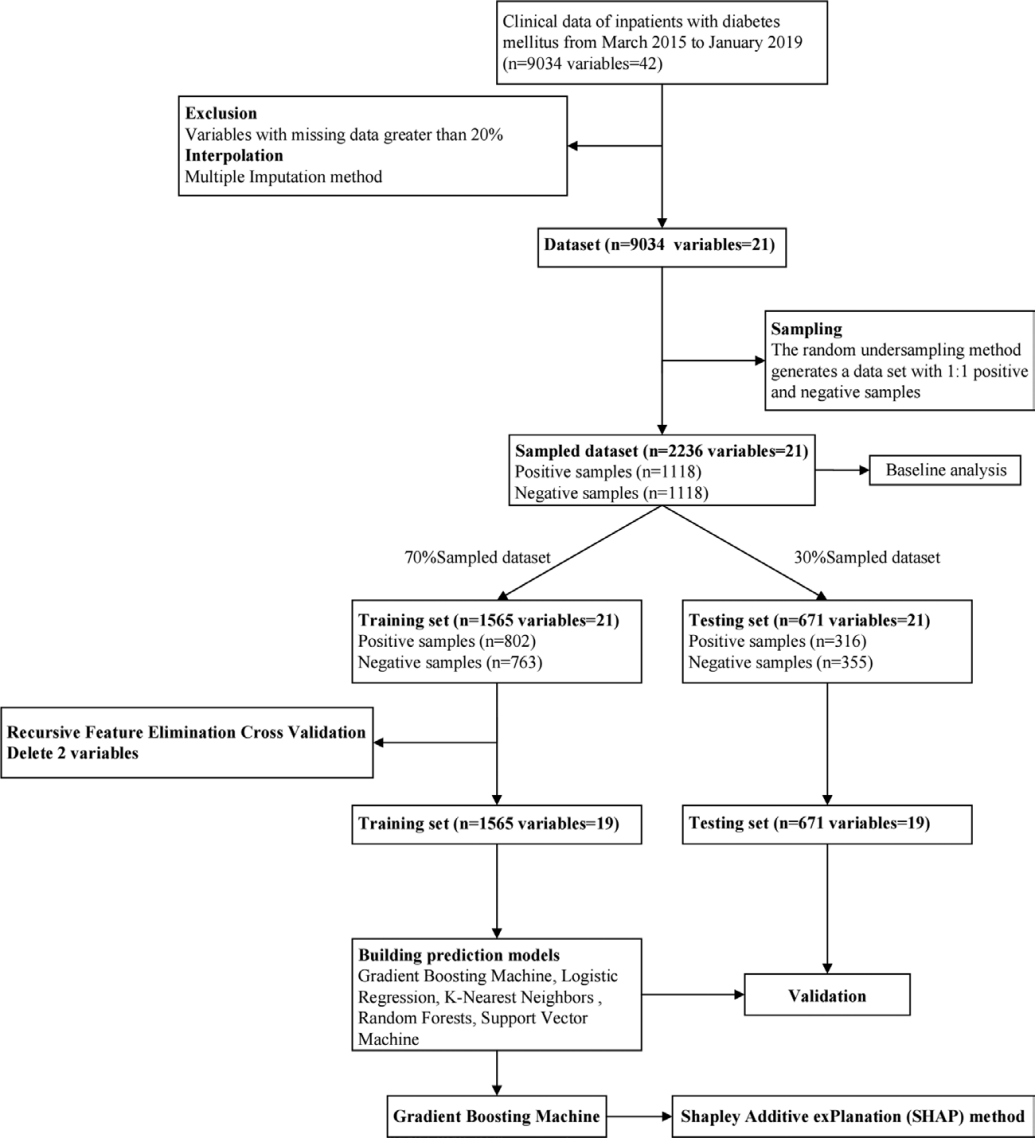
General schema for prediction model building and evaluation. The positive samples were defined as patients with DR, and negative samples were patients without DR. DR = diabetic retinopathy.

As machine learning has developed, it has become a powerful tool for predicting the risk of disease.^[9]^ For example, Support Vector Machine (SVM),^[10]^ Decision Tree,^[11]^ Gradient Boosting Machine (GBM),^[12]^ K-nearest Neighbor (KNN)^[13]^ algorithms and other machine learning technologies are used to establish corresponding disease diagnosis models. Unfortunately, most machine learning suffers from a black box problem, and the problem of interpretation becomes a bottleneck. Therefore, we screened the risk prediction variables of DR from the data of TCM and WM, and built an interpretable DR risk prediction model based on the optimized GBM and the Shapley Additive exPlanation (SHAP), providing a reliable basis for the clinical prevention and treatment.^[14]^ The model constructed in this study is shown in Figure 1.

## 2. Materials and Methods

### 2.1. Data collection

In this study, we collected the clinical data of DM from the Information Statistics Center of Hunan Provincial Health Commission and Guizhou Bailing Traditional Chinese Medicine diabetes Hospital from March 2015 to January 2019.

### 2.2. Inclusion criteria

1. Meet type 2 diabetes mellitus (T2DM) and DR diagnostic criteria.
2. Discharge diagnosis records were mined to obtain diagnostic data for specific diseases. For each variable, the first measurement record at the time of the first admission was taken.

### 2.3. Exclusion criteria

Patients with keratitis, corneal staining and other ocular conditions that interfere with fundus examination. Patients with fundus diseases other than DR.

### 2.4. Diagnostic criteria

The diagnosis criteria for T2DM followed the criteria of the American Diabetes Association “Standards of Care in Diabetes.”^[15]^ DR was diagnosed according to the Guidelines on Diabetic Eye Care of the International Council.^[16]^ All patients with diabetic fundus lesions, including mild non-proliferative DR (NPDR), were defined in the DR group (microaneurysms, intraretinal hemorrhages, venous beading (venous caliber changes consisting of alternating areas of venous dilation and constriction), intraretinal microvascular abnormalities, hard exudates (lipid deposits), and retinal neovascularization). The positive samples were defined as patients with DR, and negative samples were patients without DR.

### 2.5. Data preprocessing

#### 2.5.1. Data interpolation

In order to improve the data utilization, the missing data needed to be interpolated. Multiple imputation method was used to interpolate the individual missing data.^[17]^ Linear regression was used to estimate the value to be interpolated, and different noises were added to form 5 groups of optional interpolations.^[18]^ The data with the highest correlation coefficient in the missing data column was found as the variable to fill the prediction for regression analysis. The variable with the missing value is *y*, and the variable used for prediction filling is *x*, and the *y* value is filled according to *y* = a + b*x* + *e*, where *e* is a random number drawn from the residual. The most suitable interpolations were selected according to the value range and the comparison of the mean value before the variable was filled.

#### 2.5.2. Label assignment

We coded Qi and Yin deficiency as “1,” liver and kidney Yin deficiency as “2,” Yin and heat deficiency as “3,” and Yin and Yang deficiency as “4”, and labeled whether the patient had DR with “1” for “yes” and “0” for “no.” We also coded one-hot categorical variables for type of TCM. One-hot coding for data preprocessing of category-valued data is more reasonable for similarity or distance calculation, and more applicable to related machine model, and the usability of the model is enhanced.^[19,20]^

#### 2.5.3. Standardized processing

According to Ophthalmology of Traditional Chinese Medicine,^[21]^ and Guidelines for Traditional Chinese Medicine Prevention and Treatment of Diabetes,^[22]^ TCM syndrome types, and TCM symptoms were standardized.

#### 2.5.4. Data set division

Due to the unbalanced distribution of positive and negative samples, the method of random undersampling was used to generate data sets with 1:1. Using the train_test split function in the scikit-learn package, the data was randomly generated into a training set and a test set in a 7:3 ratio. The training set was used to train the predictive model, and the test set was used to evaluate the performance of the predictive model.

#### 2.5.5. Statistical analysis

In this study, Python (V.3.7.7) and SPSS25.0 software were used for statistical analysis of all study data. Continuous variables with skewed distribution were represented by median M (Q1, Q3), and frequency and component ratio [n (%)] were used for categorical variables. Differences between DR and non-DR patients were evaluated by using the Mann–Whitney *U* test for non-normally distributed variables, and the chi-squared test for categorical variables. All tests conducted in this study were 2-tailed, and *P* < .05 was considered to be statistically significant.^[23]^

#### 2.5.6. Feature selection

Recursive Feature Elimination with Cross-Validation (RFECV) method is used to determine the optimal variable for feature selection.^[24]^ We used 5-fold cross-validation on the training set to find the optimal number of features, and then selected different numbers of features according to the importance of features determined in the recursive feature elimination (RFE) stage of this study. For the selected feature set, the supervised learning estimator was used to calculate the average score, and the number of features with the highest average score was determined to complete feature selection.^[25]^ In the base model cross-validation, the average score was calculated first when no features were deleted. Then the scores of all combinations of n features were deleted, the scores of all combinations were averaged. And so on, until the minimum number of features was found to determine the optimal feature subset. As an automatic feature selection method, RFECV can effectively identify important features and eliminate redundancy without pre-assuming feature relationships. This method improves the performance and generalization ability of the model through cross validation and recursive elimination strategies.

In order to reflect the influence of TCM syndrome types on DR risk, the feature importance of each TCM syndrome type was calculated through GBM’s built-in interface.

#### 2.5.7. Prediction model training and validation

In this study, GBM was used to develop the predictive model. GBM is a large class of algorithms in Boosting. It was proposed by Friedman in 2002, and its idea is borrowed from the gradient descent method. GBM algorithm takes stagewise additive expansions (stepwise addition and extension) and steepest-descent minimization (steepest-descent minimization), that is, the gradient descent method is combined to achieve numerical optimization of function spaces, which can be applied to regression and classification problems. Compared with other models, it has the advantages of good integrity, strong robustness and good interpretation.^[26]^ Moreover, when the tuning time is relatively short, the accuracy of the prediction can be relatively high. To make the model more convincing, we also compared the performance of GBM with 4 other popular machine learning techniques, including Logistic Regression, KNN, Random Forest, and SVM. We reserved 30% of the data set for testing, and then performed 10-fold cross-validation on the remaining 70% of the training set. The best model obtained from the cross-validation was evaluated using the pre-reserved test set. To obtain realistic, generalizable estimates and conservative confidence intervals, each model was fitted based on the training data set, the parameters of each model were preliminarily tuned through grid searches. And the model performance was evaluated on the test data set. In this study, accuracy, F1 score, recall, precision and area under the characteristic curve (ROC-AUC) were used as criteria for comparing model performance. Given a true negative (TN) value, the values of true positive (TP), false negative, and false positive were calculated from the confusion matrix. The true positive rate (TPR) and false positive rate were used to plot the X-axis and Y-axis of the ROC-AUC curve. The specific calculation formula is presented in Eqs (1–5).

$$\begin{array}{l} Accuracy\;=\frac{TP+TN}{TP+TN+FP+FN} \end{array}$$ (1)

$$\begin{array}{l} F1\;Score\;=\frac{2\times Precision\times Recall}{Precision+Recall} \end{array}$$ (2)

$$\begin{array}{l} Recall\left(TPR\right)=\frac{TP}{TP+FN} \end{array}$$ (3)

$$\begin{array}{l} Precision=\frac{TP}{TP+FP} \end{array}$$ (4)

$$\begin{array}{l} FPR=\frac{FP}{FP+TN} \end{array}$$ (5)

## 3. Results

### 3.1. Data set

The data of 2236 T2DM inpatients including 1118 DR patients and 1118 non-DR patients, and 42 variables was extracted. Variables were deleted due to data loss exceeding 20%. Among them, the *P* values of sex, TCM syndrome type, glycosylated hemoglobin (HbA1c), low density lipoprotein (LDL), high density lipoprotein (HDL), triglyceride (TG), blood urea nitrogen, creatinine, total bilirubin, total protein (TP), globulin, albumin (ALB), aspartate aminotransferases, and γ-Glutamyltransferase were less than 0.05, which had statistical significance. The statistical analysis results are shown in Table 1.

**Table 1:** Statistically significant variable analysis results

Variables		Non-DR	DR	*P* value
Sex	Male	532 (47.6)	592 (53)	.011*
	Female	586 (52.4)	526 (47)	
TCM syndrome types	Qi and Yin deficiency	359 (32.1)	222 (19.9)	<.001**
	liver and kidney Yin deficiency	517 (46.2)	426 (38.1)	
	Yin and heat deficiency	167 (14.9)	176 (15.7)	
	Yin and Yang deficiency	75 (6.7)	294 (26.3)	
HbA1c (%)		8 (7.1, 9.9)	8.8 (7.5, 10.6)	<.001**
LDL (mmol/L)		2.67 (2.25, 3.38)	3.16 (2.54, 3.9)	<.001**
HDL (mmol/L)		1.22 (1.09, 1.42)	1.11 (0.93, 1.35)	<.001**
TG (mmol/L)		1.67 (1.4, 2.32)	2.03 (1.23, 3.04)	<.001**
BUN (mmol/L)		5.73 (4.535, 7.3)	5.36 (4.37, 6.54)	<.001**
Cr (μmol/L)		66.53 (53, 84.80)	78.56 (62.81, 94.85)	<.001**
TBIL (μmol/L)		8.9 (7.8, 13.06)	12.37 (8.44, 17.24)	<.001**
TP (g/L)		66.33 (61.5, 70.9)	70 (64.26, 75.44)	<.001**
GLB (g/L)		27.18 (25.55, 28.85)	25.28 (22.50, 27.93)	<.001**
ALB (g/L)		40 (36.3, 43.5)	44.735 (40.42, 49.36)	<.001**
AST (U/L)		19 (17, 25)	20.08 (12.84, 29.95)	.022*
GGT (U/L)		32.68 (20, 45.63)	30.66 (20.21, 42.83)	.006**

The continuous variables were expressed as the median (IQR) after the normality distribution test. The categorical variables were expressed as number (percentage).

ALB = albumin, AST = aspartate aminotransferases, BUN = blood urea nitrogen, Cr = creatinine, GGT = γ-Glutamyltransferase, GLB = globulin, HbA1c = glycosylated hemoglobin, HDL = high density lipoprotein, LDL = low density lipoprotein, TBIL = total bilirubin, TCM = traditional Chinese medicine, TG = triglyceride, TP = total protein.

* *P* < .05;

** *P* < .01.

### 3.2. Feature selection

**Figure 2.**
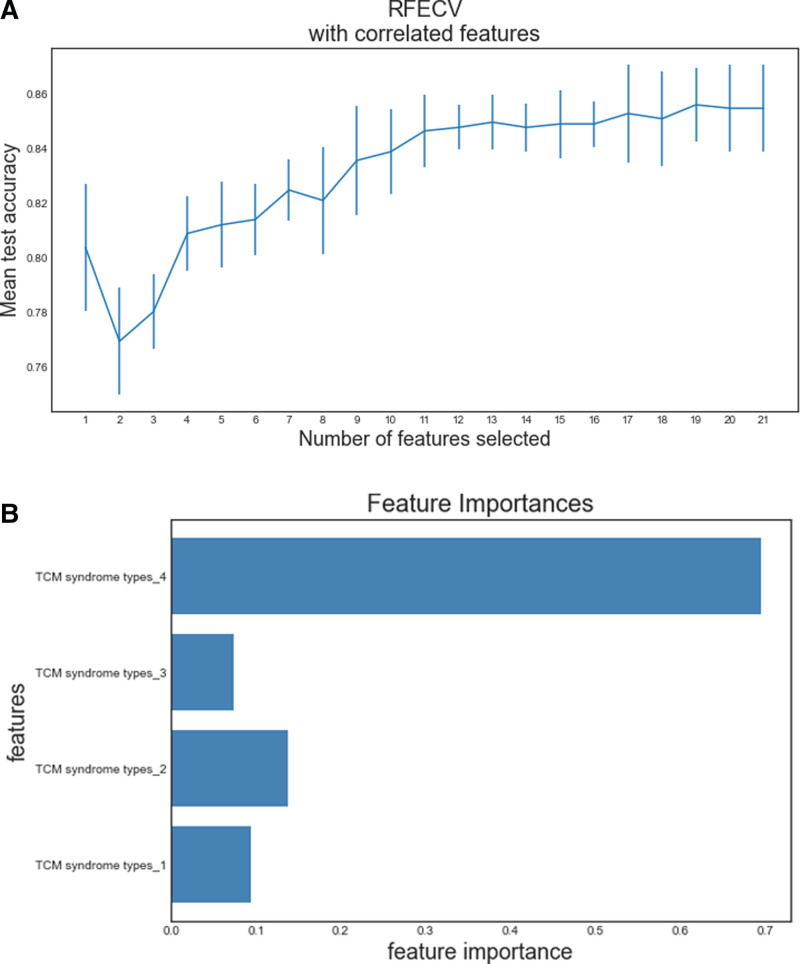
(A) Feature selection accuracy curve. (B) The feature importance of 4 TCM syndrome types. TCM = traditional Chinese medicine.

According to the results of RFECV, 19 variables such as TCM syndrome type, Age, HbA1c, LDL, HDL, total cholesterol (TC), TG, blood urea nitrogen, creatinine, total bilirubin, direct bilirubin, indirect bilirubin, TP, globulin, ALB, aspartate aminotransferases, alanine aminotransferase, alkaline phosphatase, and γ-Glutamyltransferase were selected to establish the prediction model. The features were filtered out include gender and uric acid. The change of accuracy rate with the number of variables is shown in Figure 2A. It can be clearly seen that when the number of features is 19, the accuracy of the model is the highest. Ten random_state are generated to divide the training set and the test set of the data set, and 10 repeated experiments are carried out with different training sets and test sets to prove the stability of this feature selection. The results of repeated experiments are shown in Table 2. Furtherly, we can see from Figure 2B that the characteristics of Yin and Yang deficiency syndrome and liver and kidney Yin deficiency syndrome in TCM syndrome type are of high importance. In Table 1, we can also see that Yin and Yang deficiency syndrome and liver and kidney Yin deficiency syndrome in the DR group is relatively high.

**Table 2:** Repeated experiments on feature selection

Training times	Random_state	Number of features	Accuracy
1	289	19	0.8524
2	200	16	0.8473
3	150	19	0.8594
4	433	19	0.8488
5	42	14	0.8422
6	323	19	0.8581
7	77	17	0.8447
8	267	19	0.8580
9	112	18	0.8575
10	4	19	0.8477

### 3.3. Model performance

**Figure 3.**
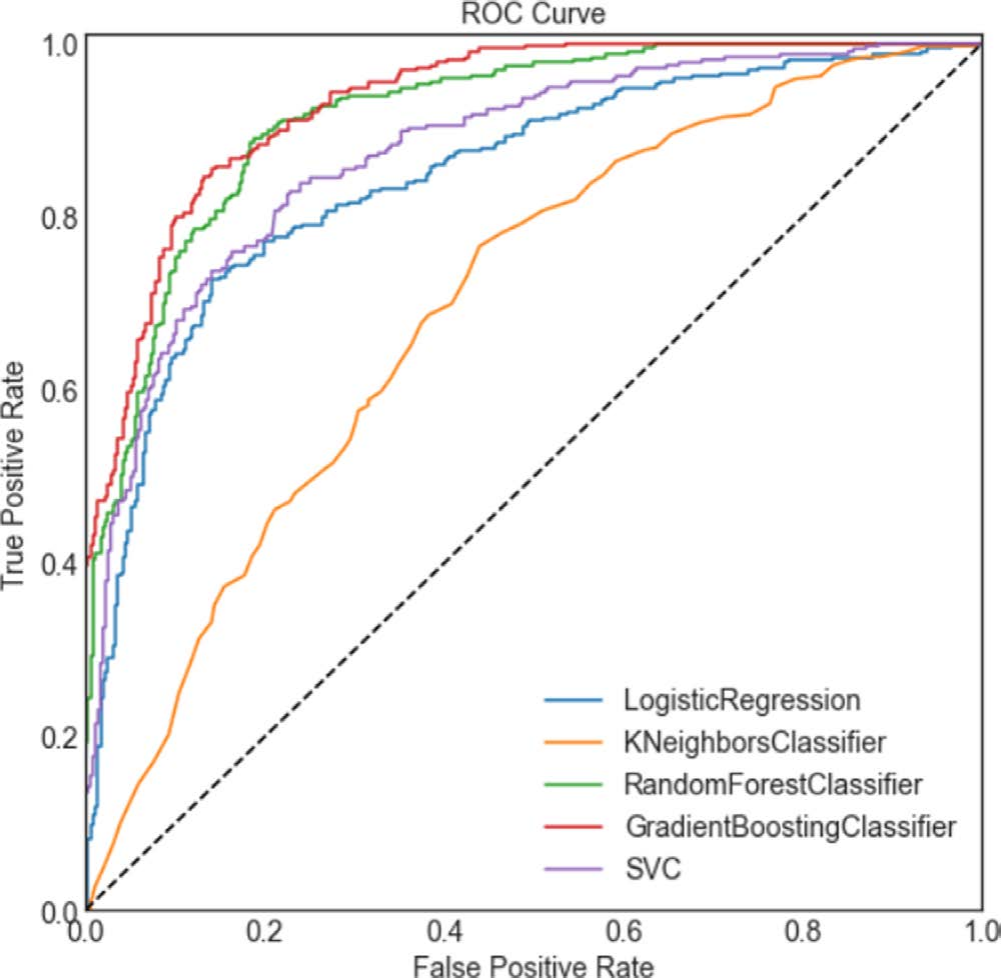
ROC curves are plotted for each individual model and the ensemble model. The dotted line represents random-guessing. ROC = receiver operating characteristic curve.

The receiver operating characteristic curve (ROC) in Figure 3 shows the overall discriminative power of the model. As can be seen from the figure, the gradient boosting classifier has the best performance with an AUC of 0.934. The Random Forest also performs very well, with an AUC of 0.918. The other 3 classifiers are significantly worse, with KNN being the worst performer, which is also showed in Table 3.

**Table 3:** Performance of prediction models in the validation set

Model	Accuracy	F1 score	Recall	Precision	AUC
LR	0.77	0.77	0.77	0.77	0.846
KNN	0.71	0.71	0.71	0.71	0.771
GBM	0.85	0.85	0.85	0.85	0.934
SVM	0.74	0.75	0.75	0.75	0.819
RF	0.83	0.83	0.83	0.83	0.918

Accuracy, F1 score, recall, precision and area under the characteristic curve (ROC-AUC) were used as criteria for comparing model performance.

GBM = Gradient Boosting Machine, KNN = K-nearest Neighbor, LR = logistic regression, RF = random forest, SVM = support vector machine.

**Figure 4.**
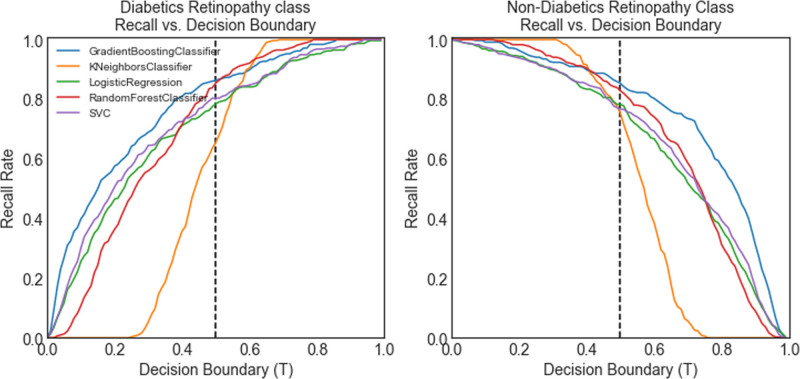
Recall vs decision boundary curves for DR and non-DR by model. DR = diabetic retinopathy.

And we can also see the recall and decision boundary curves for all models for DR and non-DR patients in Figure 4. As shown, GBM classifier has the highest performance and the KNN classifier has the worst performance. It is obvious to find that GBM classifier is the most stable, and so we choose it for our analysis.

**Figure 5.**
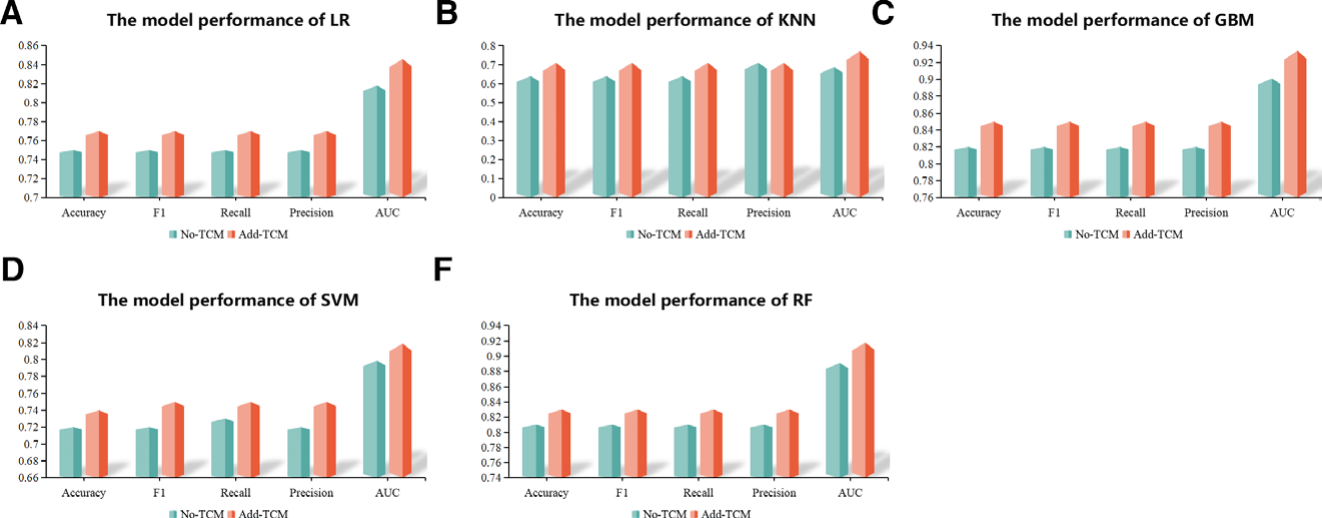
The performance of models whether included TCM syndromes or not. TCM = traditional Chinese medicine.

In addition, the results in Figure 5 show that the performance of the prediction model is improved after the addition of TCM syndrome types.

### 3.4. Model optimization

Grid search is an excellent strategy used to find the best hyperparameters as it tests for each hyperparameters within defined ranges instead of testing the combination of parameters randomly. Grid search suffers from high-dimensional spaces, but can often be easily parallelized because the hyperparameter values the algorithm works with are usually independent of each other.^[27,28]^

It is therefore used a grid search to optimize the model step by step by setting a range of hyperparameters. By exhaustive the specified parameter combinations and evaluating the performance of each set of parameters on the validation set, the best performing parameter combination is selected. The criterion of grid search is to find the combination of parameters that performs best on the validation set. After obtaining the optimal parameters, the step size was halved to prevent overfitting and the maximum number of iterations was doubled to increase the generalization of the model. The optimized model is seen in Table 4, where the accuracy is improved from 0.85 to 0.88 and the AUC is improved from 0.934 to 0.958, which improves the performance.

**Table 4:** Performance metrics for the optimized model

Model	Accuracy	F1 score	Recall	Precision	AUC
GBM	0.85	0.85	0.85	0.85	0.934
Optimized GBM	0.88	0.88	0.88	0.88	0.958

GBM = Unoptimized Gradient Boosting Machine, Optimized GBM = Gradient Boosting Machine Optimized by Grid Search.

### 3.5. DR influencing factors assessment

**Figure 6.**
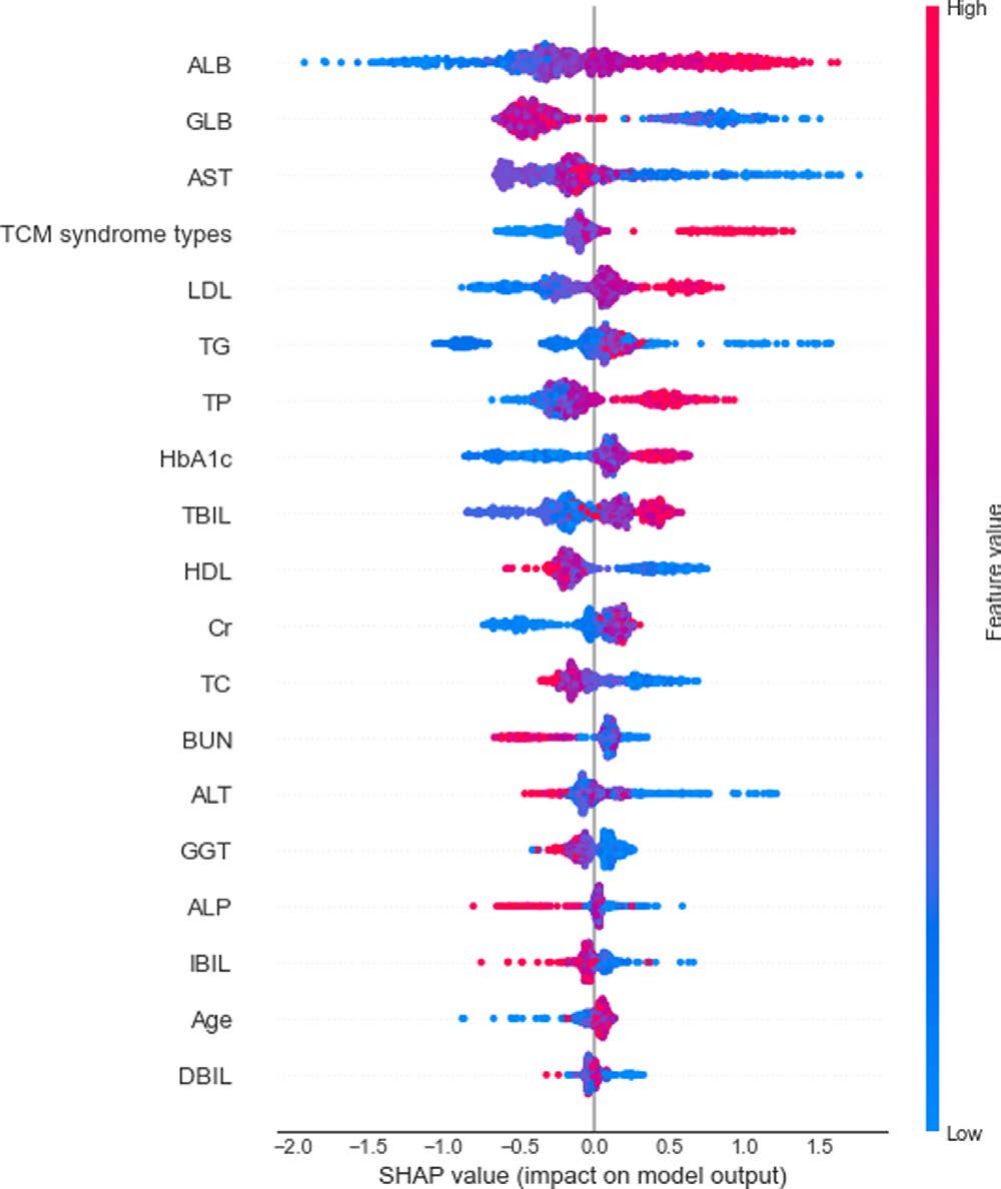
SHAP summary plot of the GBM classifier. GBM = Gradient Boosting Machine, SHAP = Shapley Additive explanation.

In order to determine the influencing factors of DR, we constructed the SHAP summary graph of the GBM classifier (Fig. 6). The position on the horizontal axis is determined by each Shapley value. Color represents eigenvalues (red high, blue low), and color allows us to match how changes in eigenvalues affect changes in risk. The overlap points are dithered in the vertical axis direction, so we can understand the Shapley value distribution for each feature, and the features are ranked according to their importance. As shown in the figure, ALB, TCM syndrome type, LDL, TG, TP, and HbA1c were the main factors leading to the increased risk of DR, which is basically consistent with the characteristic importance results obtained by RFECV. Horizontally, the sample distribution of ALB is relatively scattered, which means that the influence of this feature is greater. Longitudinally, SHAP values corresponding to features such as ALB, TCM syndrome type, LDL, TG, TP, and HBA1c were greater than 0, indicating that these features were important factors causing DR.

**Figure 7.**
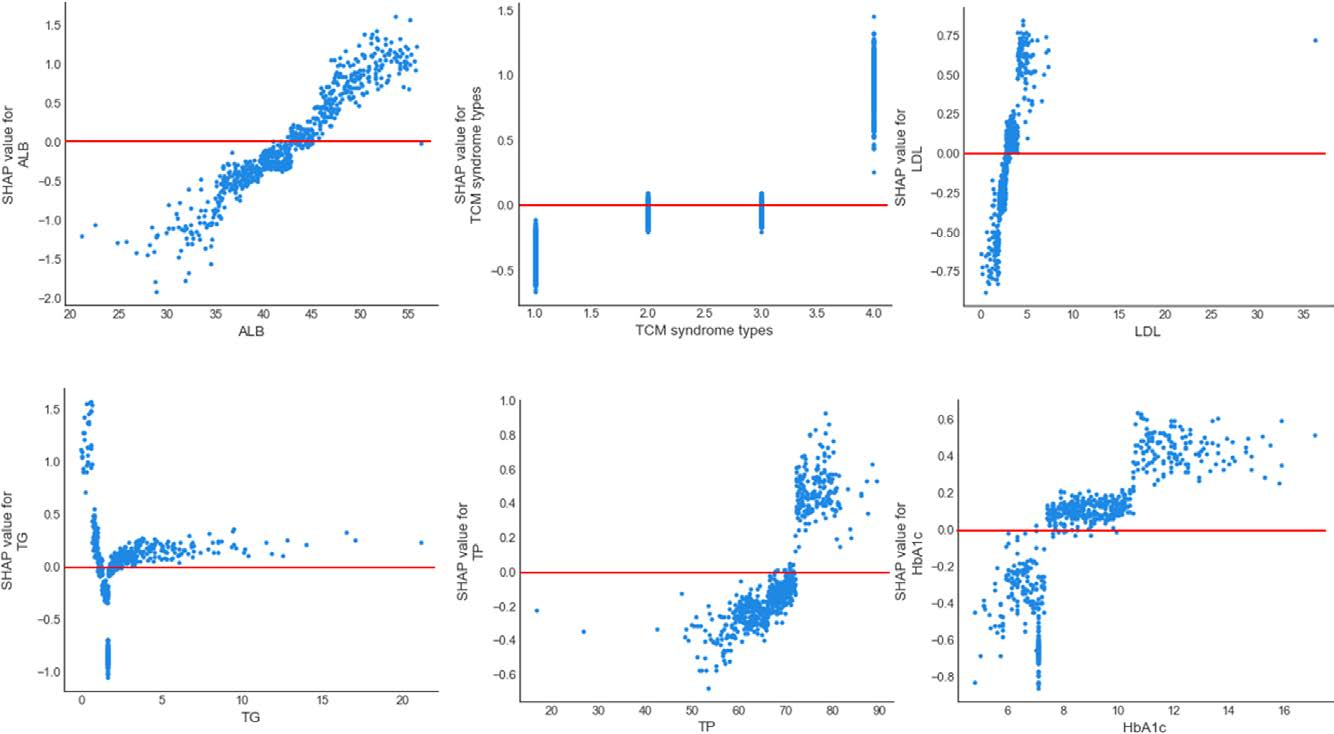
SHAP dependence plot of the GBM classifier. GBM = Gradient Boosting Machine, SHAP = Shapley Additive explanation.

The SHAP dependency graph shows the influence of a single feature on the output of the GBM classifier (Fig. 7). ALB value greater than 45 g/L, Yin and Yang deficiency syndrome, LDL value greater than 5 mmol/L, TG greater than 2.5 mmol/L, TP greater than 80 g/L, HbA1c greater than 8% were associated with increased risk of DR.

## 4. Discussion

DR is one of the leading causes of vision loss worldwide. As the prevalence of DM increases, there is an urgent need to provide targeted guidance on the prevention and management of DR, reflecting the need to analyze the factors influencing DR.^[23,29]^ According to TCM masters, the most crucial factor in this disease is the simultaneous occurrence of Qi deficiency and Yin deficiency and stagnation. Prolonged DM disease weakens the body and the kidneys gradually lose their essence and energy, thus exacerbating the deficiency of Qi and blood. Over time, blood clotting can lead to retinal neovascularization.^[30]^ The overall concept of TCM and evidence-based treatment has achieved significant therapeutic effects in guiding its clinical treatment, and compared with WM treatments, no obvious toxic side effects have been observed.^[31,32]^

The integration of ophthalmology and machine learning models can change the diagnosis of disease patterns, reducing manpower, while improving efficiency and accuracy. What’s more, there are no specific symptoms in the early stages of DR, making it difficult to diagnose accurately. The large number of DM groups, uneven distribution of medical resources, high screening costs, and high financial pressure on patients are all obstacles to this. Therefore, the establishment and selection of a suitable predictive model is of great significance for reducing the incidence, early diagnosis and prevention of DR.

In recent years, many scholars have constructed research models about DR. Yang et al^[5]^ tested referable DR in diabetic patients by comparing 8 machine learning models. The XGBoost classifier showed the best performance with an AUC of 0.816, which was verified in independent populations. Oh et al^[6]^ combined the sparse learning model to conduct DR risk assessment, and used the minimum absolute contraction and selection operator combined with the Bayesian information standard to evaluate the internal verification group, and obtained the best AUC, accuracy, sensitivity and specificity, which were 0.81, 0.736, 0.774, and 0.727, respectively. Ogunyemi et al^[7]^ constructed a set classifier for predicting DR, and the optimal AUC, accuracy, sensitivity and specificity of the test set were 0.71, 0.735, 0.654, and 0.569, respectively. Tsao et al^[8]^ divided the clinical data of 536 patients in Taiwan into a training set and a validation set (ratio: 80:20), and compared the performance of 4 models (support vector machine, decision tree, ANN and logistic regression) in DR detection, and found that the AUC of support vector machine was 0.839. The performance of the methods proposed in the above research is summarized in Table 5. Compared with the above research, the accuracy and AUC obtained by the model adopted in this study have been improved to some extent on the test set.

**Table 5:** Model evaluation of relevant research

Approaches	Data sets	Accuracy	Sensitivity	Specificity	AUC
Yang et al	China	0.796	0.796	0.799	0.816
Oh et al	South Korea	0.736	0.774	0.727	0.810
Ogunyemi et al	United States	0.735	0.654	0.569	0.710
Tsao et al	China	0.795	0.933	0.724	0.839

In this study, SHAP values are conducive to making the output of the GBM classifier model clinically interpretable. Through SHAP method, the most critical risk factors and critical values of risk factors for DR can be found out. This provides more targeted recommendations for the treatment and management of DM patients and has important implications for reducing the number of patients with DR. GBM classifier shows that ALB is the most important risk factor for DR, followed by TCM syndrome type. LDL, TG, TP and HbA1c may also increase the risk of DR.

Changes in ALB levels may be linked to inflammatory responses and oxidative stress, which may accelerate the development of the DR. In addition, ALB is a small molecular weight protein that can pass through the glomerular filtration membrane, and almost all of the normal ALB is reabsorbed by the proximal convoluted tubules. If the glomerular lesions occur, ALB filtration excess exceeds the maximum tubule reabsorption, and urine ALB content increases, and the degree of increase is positively correlated with the degree of glomerular injury. The appearance of proteinuria in DM patients indicates the possibility of extensive microangiopathy, predicts the occurrence of diabetic nephropathy, and is also related to the occurrence of other complications of microangiopathy, and therefore has a very significant correlation with DR.^[33]^ Based on the theoretical basis of “liver and kidney homology” in TCM^[34]^ and the pathophysiological factors of diabetic microangiopathy in WM,^[35]^ some researchers have proposed the treatment idea of “eye and kidney co-treatment,”^[36]^ which reflects the certain relationship between kidney and DR.

TP has a SHAP value greater than 0, which indicates that its elevation leads to an increased risk of DR. When the human liver is diseased, such as inflammation of the liver cells, it will lead to the increase of TP, which is a reflection of the level of liver function. However, there is a lack of research on the mechanism of liver function on DR, and the mechanism of action is still unclear. However, there has long been a precedent for treating DR by invigorating kidney and clearing liver and eliminating blood stasis and improving eyes in China. According to TCM, T2DM is mostly caused by “excessive injury of 7 emotions and excessive exhaustion, and its pathogenesis is fundamentally Yin deficiency and heat, which damages the Yin of liver and kidney for a long time, and cannot be carried on the eye network, the eye is displaced and nourishing, and it is appropriate to treat the kidney and clearing liver, eliminating blood stasis and improving eyes.”^[37]^ Indirectly, the level of liver function may also affect the incidence of DR, which provides a new way to study the pathogenesis of DR.

For TCM syndrome types, the basic pathogenesis of DR is Yin deficiency. TCM believes that the pathogenesis of DR is mostly caused by lack of innate endowments and loss of post-natal recuperation, which leads to Yin deficiency, Yin deficiency of liver and kidney, blood accumulation into stasis, and failure of Qi to rise to the eye.^[38]^ More and more studies have shown that DR is related to the inflammatory response caused by inflammatory mediators, and chronic low-grade inflammation can cause retinal vascular system damage.^[39]^ At the same time, some scholars have shown that phlegm, stasis and deficiency are closely related to chronic inflammation and its inflammatory factors.^[40,41]^ Studies have shown that TCM syndrome type is related to DR staging, and the stages increase gradually with the evolution of DR pathogenesis. In non-DR and mild NPDR stage, Qi-Yin deficiency syndrome accounted for the highest proportion (80.6%). In the moderate and severe NPDR stage, the main proportion of liver and kidney deficiency was 55.6%; and in proliferative DR stage, Yin-Yang deficiency syndrome accounted for 68.8%.^[42]^ From the perspective of the development law of DR, the Diabetes Branch of the Chinese Society of Traditional Chinese Medicine^[43]^ summarized the development of DR from “Qi and Yin deficiency” to “liver and kidney Yin deficiency” to “Yin and Yang deficiency.” In this study, it can be seen from Figure 7 that Yin and Yang deficiency and liver and kidney Yin deficiency have a greater impact on DR. Other studies^[22]^ have pointed out that both obese and non-obese DM can lead to liver and kidney Yin deficiency or kidney Yin and Yang deficiency, various chronic complications, and death in severe cases. This indicates that TCM syndrome type has certain reference value for predicting DR. Relevant studies have shown that abnormal lipid metabolism is one of the main risk factors for the occurrence of DR. Compared with DM patients without DR, the lipid level of patients in the DR group is significantly beyond the normal range.^[44]^ Abnormal lipid metabolism leads to hemodynamic disturbance, oxidative injury of endothelial cells and changes in lipid structure of cell membrane. It leads to pathological changes such as retinal microcirculation disturbance, tissue hypoxia, unstructured angiogenesis, hemorrhage, exudation, edema, etc., which promote the occurrence and development of DR.^[45]^ However, the pathogenesis of DR caused by blood lipids has not been fully elucidated. In this study, the SHAP value of LDL and TG was greater than 0, which promoted the occurrence of DR. The SHAP value of HDL was less than 0, indicating that it may be a protective factor for DR.

HbA1c is an important index to evaluate the average blood glucose level of the body, which can reduce blood oxygen level by affecting the physiological function of hemoglobin, increase the viscosity of red blood cells, lead to retinal hypoxia, and lead to microvascular diseases of the visual retinal membrane.^[46]^ High level HbA1c can also induce oxidative stress, which triggers and aggravates retinal damage.^[47]^ And it can lead to vascular endothelial injury and decrease microcirculation, resulting in ischemic hypoxic injury of ocular nerves, thereby reducing vision and causing DR.^[48]^ It has shown high accuracy in the diagnosis of DR,^[49]^ and is also considered as an indicator of good diagnostic accuracy in early complications of DM.^[50]^ At present, HbA1c is generally considered to be a risk factor for DR. Alvarez Ramos et al^[51]^ found that the risk of DR increased by 58% for every percentage point increase in HbA1c. There is a linear relationship between HbA1c and DR.^[52]^ Recent studies^[53]^ have found that the interaction between glucose metabolism and lipid metabolism in DR plays an important role in the occurrence and development of DR, but the relationship between the 2 remains unclear. Other studies have shown that both HbA1c level and DM have a certain impact on liver lesions.^[54]^

This study has several advantages. First, all the variables come from professional hospitals and information statistics centers, and the data are authentic and reliable. The patients’ daily physical conditions are clearly recorded in the cases, including tongue, pulse, sweating, sleep status, defecation status, etc., which is very important for TCM diagnosis. Secondly, we use RFECV to select the optimal variable combination, which reduces the time of selecting variables and gets more accurate results. Finally, the ranking of feature importance may provide insights into the prevention of DR.

However, there are several limitations of this study. The experimental data have some limitations and come only from hospitals in Hunan and Guizhou provinces in China, which may be subject to local influences. In addition, the number of features included in this study was small, and DM course was not included due to the absence of real-world data. This experimental hyperparameter tuning uses only one method, grid search. But in the future, more data and features can be combined and multiple tuning methods can be used to optimize the model to achieve the goal of obtaining a better performing model.

## 5. Conclusion

It can be concluded that TCM syndrome type, ALB, LDL, TG, TP, HbA1c were strongly associated with an increased risk of DR. The risk prediction model of integrated TCM and WM based on machine learning is more effective for DR. This provides new indicators for risk prediction of DR and new ideas for screening and prevention of DR.

## Author contributions

**Conceptualization:** Li Xiao.

**Data curation:** Lixuan Tang, Wenxuan Kuang.

**Formal analysis:** Li Xiao.

**Investigation:** Ying Deng.

**Methodology:** Li Xiao, Yijing Yang.

**Software:** Li Xiao, Lixuan Tang.

**Supervision:** Jing Lu, Qinghua Peng, Junfeng Yan.

**Writing – original draft:** Li Xiao.

**Writing – review & editing:** Lixuan Tang, Jing Lu, Qinghua Peng, Junfeng Yan.
